# Characterisation of Cyanobacterial Bicarbonate Transporters in *E. coli* Shows that SbtA Homologs Are Functional in This Heterologous Expression System

**DOI:** 10.1371/journal.pone.0115905

**Published:** 2014-12-23

**Authors:** Jiahui Du, Britta Förster, Loraine Rourke, Susan M. Howitt, G. Dean Price

**Affiliations:** Plant Science Division, Realizing Increased Photosynthetic Efficiency (RIPE) Network, Research School of Biology, Linnaeus Building #134, Australian National University, Canberra, ACT 2601, Australia; University of New South Wales, Australia

## Abstract

Cyanobacterial HCO_3_^-^ transporters BCT1, SbtA and BicA are important components of cyanobacterial CO_2_-concentration mechanisms. They also show potential in applications aimed at improving photosynthetic rates and yield when expressed in the chloroplasts of C3 crop species. The present study investigated the feasibility of using *Escherichia coli* to assess function of a range of SbtA and BicA transporters in a heterologous expression system, ultimately for selection of transporters suitable for chloroplast expression. Here, we demonstrate that six β-forms of SbtA are active in *E. coli*, although other tested bicarbonate transporters were inactive. The *sbtA* clones were derived from *Synechococcus sp*. WH5701, *Cyanobium sp*. PCC7001, *Cyanobium sp*. PCC6307, *Synechococcus elongatus* PCC7942, *Synechocystis sp*. PCC6803, and *Synechococcus sp*. PCC7002. The six SbtA homologs varied in bicarbonate uptake kinetics and sodium requirements in *E. coli*. In particular, SbtA from PCC7001 showed the lowest uptake affinity and highest flux rate and was capable of increasing the internal inorganic carbon pool by more than 8 mM relative to controls lacking transporters. Importantly, we were able to show that the SbtB protein (encoded by a companion gene near *sbtA*) binds to SbtA and suppresses bicarbonate uptake function of SbtA in *E. coli*, suggesting a role in post-translational regulation of SbtA, possibly as an inhibitor in the dark. This study established *E. coli* as a heterologous expression and analysis system for HCO_3_^-^ transporters from cyanobacteria, and identified several SbtA transporters as useful for expression in the chloroplast inner envelope membranes of higher plants.

## Introduction

Due to projections in global population growth, there have been calls for a near doubling of global food production by 2050 [1], [2]. To meet this demand, scientists are exploring numerous genetic engineering strategies to increase crop yields by improving photosynthesis, particularly by increasing photosynthetic rates and/or water-use efficiency in crops. In C3 crop plants the current level of atmospheric CO_2_ is sub-optimal for maximal photosynthetic performance, with the competing oxygenase reaction of the primary carboxylase, Rubisco, accounting for around 30% of theoretical loss to photosynthetic CO_2_ fixation capacity [3]. Field studies have shown that elevated CO_2_ levels can increase photosynthetic rates and crop yields [4], [5]. This suggests that strategies aimed at raising CO_2_ levels in the chloroplast may be a useful approach. Recently, one multiple-stage approach to raise CO_2_ levels in the chloroplasts of crops was proposed based on the CO_2_-concentrating mechanism (CCM) components of photosynthetic bacteria cyanobacteria [6]–[8]. Two key features of the cyanobacterial CCM are the use of active transport systems for uptake of inorganic carbon (Ci, including CO_2_ and HCO_3_^-^) and the elevation of CO_2_ levels within unique protein micro-compartments, called carboxysomes, which are packed with the Rubisco enzyme [9], [10].

Cyanobacterial Ci uptake systems in model species such as *Synechococcus elongatus* PCC7942 and *Synechocystis sp*. PCC6803 are composed of two known active CO_2_ uptake systems and up to three known HCO_3_^-^ transporters. They have different substrate affinities, maximal rates and energisation, which may provide different advantages for expression in C3 chloroplasts. Three HCO_3_^-^ transporters, including BCT1, SbtA and BicA, have been identified so far [6]. Among these transporters, BCT1 is a four-subunit ATP-binding cassette (ABC) transporter while SbtA and BicA are both single subunit transporters. SbtA and BicA have been initially chosen as candidates to be expressed in crops because they are both encoded by a single gene and therefore much easier to manipulate.

The SbtA transporter is a high affinity and low flux rate HCO_3_^-^ transporter, for example, SbtA affinity determined in *Synechococcus* PCC7002 has a K_m_[HCO_3_^-^] of about 2 µM [11]. SbtA is Na^+^-dependent, requiring about 1 mM Na^+^ for half-maximal HCO_3_^-^ transport activity [12]. The gene encoding SbtA, *sbtA*, is inducible under limiting Ci conditions. SbtA has 10 transmembrane domains, in a 5+5 inverted orientation with the N- and C-termini extra cellular and the two halves of the transporter are separated by an intracellular loop of variable size [9]. Curiously, a gene, *sbtB*, encoding a small soluble protein (SbtB) is found to exist in the same operon as *sbtA* in some cyanobacterial species and nearby in others [13]. The *sbtB* gene is also expressed under Ci-limited conditions in *Synechocystis sp*. PCC6803 and *Synechococcus elongatus* PCC7942 [14], [15]. The co-occurrence suggests that SbtB may be functionally related to SbtA, possibly as a regulator, but this has not yet been investigated.

The BicA transporter can support a high photosynthetic flux rate, although it has a relatively low transport affinity with a K_m_ [HCO_3_^-^] of 75–350 µM [11]. BicA is also Na^+^-dependent and requires, similar to SbtA, about 1 mM Na^+^ for half-maximal transport activity [11]. BicA is predicted to be a single subunit transporter and belongs to the SulP/SLC26A protein family. Topology mapping and threading to a known crystal structure of related proteins strongly support 14 transmembrane domains with the N- and C- termini in the cytoplasm [16].

Although both transporters seem good candidates for expression in chloroplasts, a complicating fact is that both undergo some form of post-translational regulation because Ci uptake in cyanobacteria appears to be inactive in the dark [8]. Therefore it is unclear whether these transporters will be active when expressed in crops. In fact, recently, BicA expressed in the tobacco chloroplasts appeared to be inactive [17]. A better understanding of their regulation may allow manipulation of their regulatory systems or co-expression of activators, overcoming possible problems with inhibition. To this end we needed to develop a heterologous system for selection and characterisation of transporters which are active in non-cyanobacterial environment.

Both SbtA and BicA are widely distributed within cyanobacterial species, resulting in the availability of many different homologs to screen for ease of expression and regulatory properties [13]. Cyanobacteria are divided into two phylogenetic groups based on their Rubisco and carboxysomes phylogenies, referred to as α-cyanobacteria (largely oceanic) and β-cyanobacteria (freshwater, estuarine), based on their Rubisco and carboxysomes phylogenies [18]. In general, α-cyanobacteria have only a minimal CCM and possess fewer constitutively expressed Ci transporters while the β-cyanobacteria have much more diverse range of Ci transporters [13], [18]. In addition to generally defined α- and β- cyanobacteria, some strains of α-cyanobacteria (typically *Cyanobium* strains) have been classified as transitional strains since they have moved to freshwater estuarine environments and gained genes, including Ci uptake systems, from β-cyanobacteria, probably through horizontal gene transfer [13]. This conclusion was supported by the similarity in kinetic response of external Ci by the transitional strain, *Cyanobium* spp. PCC7001, to β-cyanobacteria *Synechococcus elongatus* PCC7942 [19].

There also exist sequence differences within each transporter family that correlate with the cyanobacterial classification. For example, the loop connecting helix 5 and 6 of SbtA in the transitional strains is much shorter than the loop in β-cyanobacteria [9]. It has been suggested a partial deletion in this region may have occurred at the time of horizontal gene transfer [13]. The functional importance of the helix 5/6 loop remains to be determined, but it may have a regulatory role or a link with HCO_3_^-^ transport affinity. To date, transporters shown to have HCO_3_^-^ uptake activity are mostly from α-cyanobacteria, including SbtA from *Synechocystis sp*. PCC6803 [12] and *Synechococcus sp*. PCC7002 [11], BicA from *Synechococcus sp*. PCC7002 [11] and BCT1 from *Synechococcus elongatus* PCC7942 [20]; the only α-cyanobacterial HCO_3_^-^ transporter analysed was BicA from *Synechococcus* WH8102 [11].

The aim of the present study was to investigate the expression of a range of SbtA and BicA transporters in *E. coli* for further characterisation. *E. coli* is considered a good candidate for study of cyanobacterial HCO_3_^-^ transporters for two reasons. First, there already exists a high CO_2_-dependent *E. coli* mutant (EDCM636) that may allow positive selection of HCO_3_^-^ transporters. *E. coli* possesses two carbonic anhydrases, Can and CynT [21]. CynT is normally not expressed, so the *can* gene knockout lacks carbonic anhydrase (CA) activity, and *E. coli* can grow in high CO_2_ but not in normal air due to lack of internal HCO_3_^-^ supply [21]. Since HCO_3_^-^ is required for anaplerotic metabolism, expression of an active HCO_3_^-^ transporter should theoretically restore growth of CA-deficient *E. coli* in air and therefore could allow positive screening of HCO_3_^-^ transporters. Second, topology mapping of BicA and SbtA [9], [22] has determined that both full length transporters are expressed in the *E. coli* plasma membrane, although uptake function was not previously examined. A potential drawback of utilising *E. coli* as a heterologous system for quantitative HCO_3_^-^ transport analyses is that the CO_2_ generated by cell respiration may introduce errors in determining the kinetics of ^14^C-HCO_3_^-^ uptake by these transporters which is further discussed in the context of the results presented.

In this paper, we demonstrate that six SbtA homologs are active in our *E. coli* expression system, three from the transitional strains, *Synechococcus sp*. WH5701 (SbtA5701), *Cyanobium* spp. PCC7001 (SbtA7001) and *Cyanobium sp*. PCC6307 (SbtA6307) and three from β-cyanobacteria, *Synechococcus elongatus* PCC7942 (SbtA7942), *Synechocystis sp.* PCC6803 (SbtA6803), and *Synechococcus sp*. PCC7002 (SbtA7002). Importantly, this is also the first experimental evidence that four SbtA homologs, SbtA7942, SbtA6307, SbtA5701 and SbtA7001, are in fact functional HCO_3_^-^ transporters. Additionally, our analyses begin to define a role for SbtB as a post-translational regulator of SbtA, potentially via direct interaction of these two proteins.

## Results

### Screening for putative HCO_3_^-^ transporters that are functional in *E. coli*

**Figure 1:**
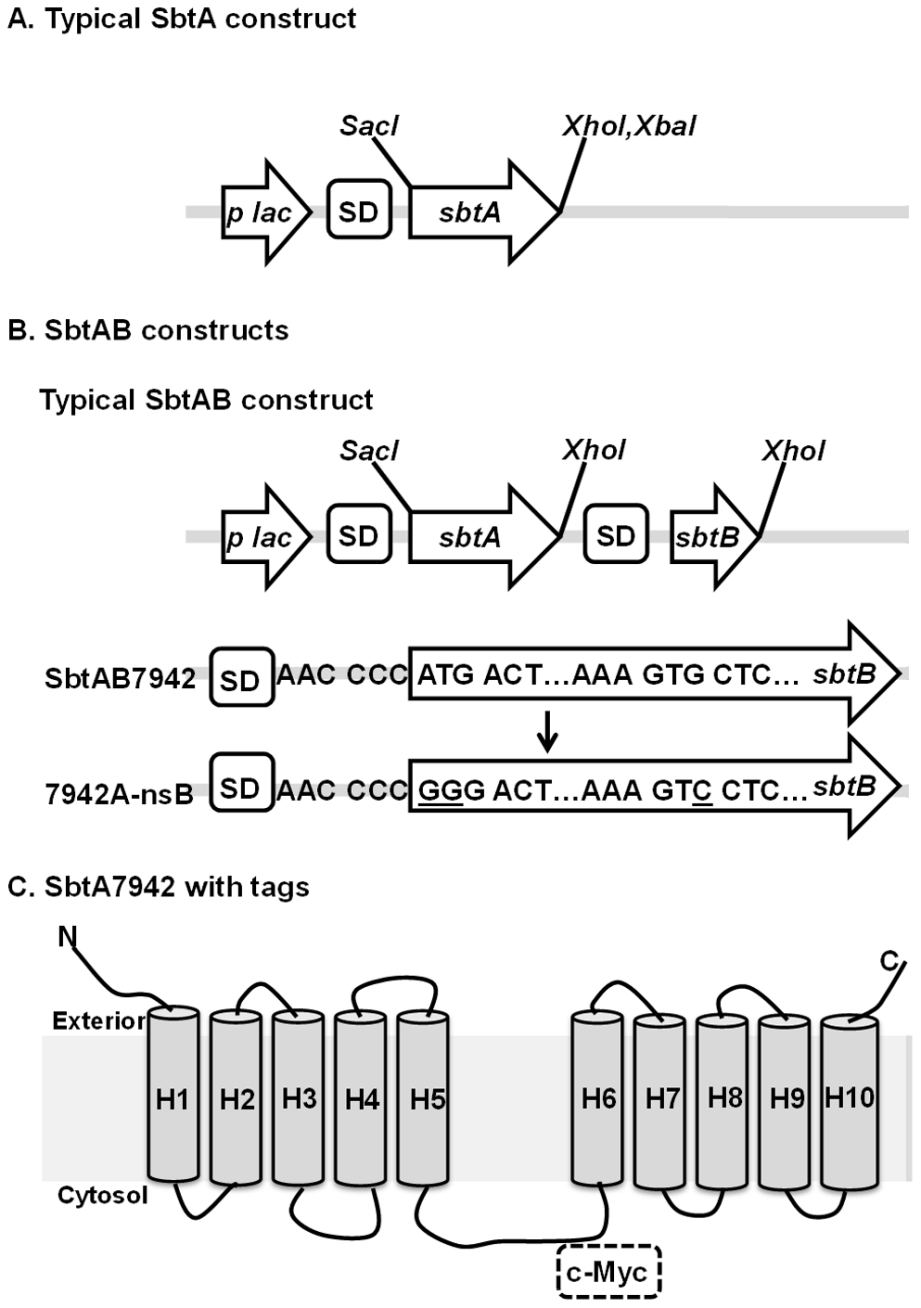
Construct designs involved in characterisation of SbtA transporters. In all constructs, expression of target proteins was driven by the *lac* promoter on plasmid *pSE2*. A. Schematic of typical SbtA constructs shown in Table 4. B. Schematic of typical SbtAB constructs shown in Table 4. In order to generate 7942AnsB, the start codon of *sbtB* (ATG) in SbtAB7942 construct was replaced with (GGG) to form a *SmaI* site and a later GTG -valine 41 bp downstream of the start codon was replaced with GTC-valine. In this way, expression of *sbtB* is completely abolished in 7942AnsB. C. Illustrated location for the c-Myc tag in 7942AMyc. SD, Shine-Dalgarno sequence.

A number of putative HCO_3_^-^ transporters were screened in *E. coli* for HCO_3_^-^ uptake activity (Table 1). The respective cDNA sequence of each transporter was cloned into the *pSE2* vector as illustrated in Fig. 1. The threshold for significant activity was set at a 2-fold increase in HCO_3_^-^ uptake compared to an empty *pSE2* vector (negative control) at pH 8. All putative transporters belong to families where some members had been proven to have HCO_3_^-^ transport activity, including BCT1 [20], BicA7002 [11], SbtA6803 [12], and SbtA7002 [11]. We also included uncharacterised SbtA and BicA homologs in our analysis. Note that SbtA proteins from oceanic α-cyanobacteria were excluded since initial testing of SbtA from *Prochlorococcus* MED4 (CCMP1986) in our cyanobacterial expression system [11] had revealed no detectable transport activity. Two screening methods were used: HCO_3_^-^ uptake experiments and complementation of the CA-deficient strain EDCM636.

**Table 1:** HCO_3_^-^ transporters tested for function in *E. coli*.

Transporter	Derivation Strain				HCO_3_^-^ Uptake
**ATP-binding Cassette (ABC) Family**					
BCT1	*Synechococcus* spp. PCC7942				No
**Sulphate Permease (SulP) Family**					
BicA7002	*Synechococcus* spp. PCC 7002				No
BicA5701	*Synechococcus* spp. WH5701				No
BicA1 7001	*Cyanobium* spp. PCC7001				No
BicA2 7001	*Cyanobium* spp. PCC7001				No
*Vibrio* SulP	*Vibrio parahaemolyticus*				No
**Sodium Dependent Bicarbonate Transporter (SBT) Family**					
SbtA7001 (SbtA1)	*Cyanobium* spp. PCC7001				Yes
SbtA7942	*Synechococcus* spp. PCC7942				Yes
SbtA6803	*Synechocystis* spp. PCC6803				Yes
SbtA7002	*Synechococcus* spp. PCC 7002				Yes
SbtA6307 (SbtA1)	*Cyanobium* spp. PCC6307				Yes
SbtA5701 (SbtA1)	*Synechococcus* spp. WH5701				Yes
*Labrenzia* SbtA-like	*Labrenzia alexandrii DFL-11*				No

A number of known and putative HCO_3_^-^ transporters were tested in *E. coli* DH5α for potential H^14^CO_3_^-^ uptake activity measured by the silicon oil centrifugation-filtration assay.

Expression of all SbtA homologs, with the exception of the *Labrenzia* SbtA-like transporter, facilitated enhanced HCO_3_^-^ uptake in *E. coli* while none of the other potential bicarbonate transporter appeared to increase HCO_3_^-^ uptake of *E. coli* (Table 1), strongly suggesting that all tested cyanobacterial SbtA homologs were able to transport HCO_3_^-^ and to function in the heterologous *E. coli* system.

**Figure 2:**
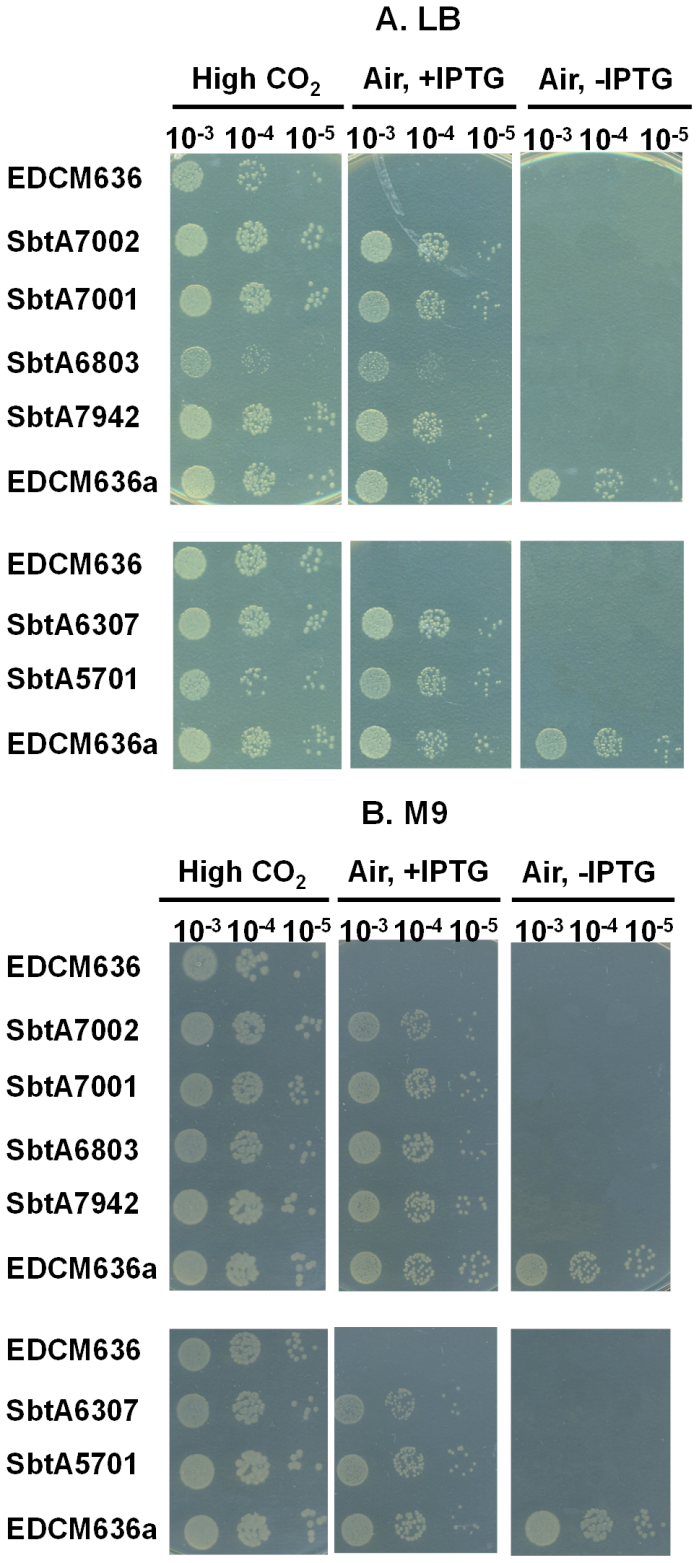
Complementation of the EDCM636 mutant by expression of various SbtA clones. Complementation of CA-deficient strain EDCM636 expressing one of the six SbtA clones, or empty vector (*pSE2*) as a control. A strain with empty *pSE2*, EDCM636a, was selected for expressed CA. EDCM636a was used as a positive control. The top panel was for growth in LB media; bottom panel was for growth in M9 media.

Consistent with the uptake data, growth of EDCM636 in air was complemented only by expression of cyanobacterial SbtA homologs. There was no obvious difference in the growth of EDCM636 in the presence of kinetically different SbtA homologs, suggesting that they were all capable of supplying enough HCO_3_^-^ for cell growth at room temperature (Fig. 2).

### Sodium dependency of HCO_3_^-^ uptake by SbtA homologs

As *Synechocystis* PCC6803 SbtA is Na^+^ dependent [12], we expected that this would also be the case for other SbtA homologs. Uptake activities of all SbtA species were stimulated by addition of NaCl, but not by comparable addition of KCl (S1 Fig.), indicating that these SbtA homologs are Na^+^ dependent in *E. coli*. The sodium dependence of each transporter was characterised in detail, ensuring other kinetic properties were analysed without Na^+^ limitation.

**Figure 3:**
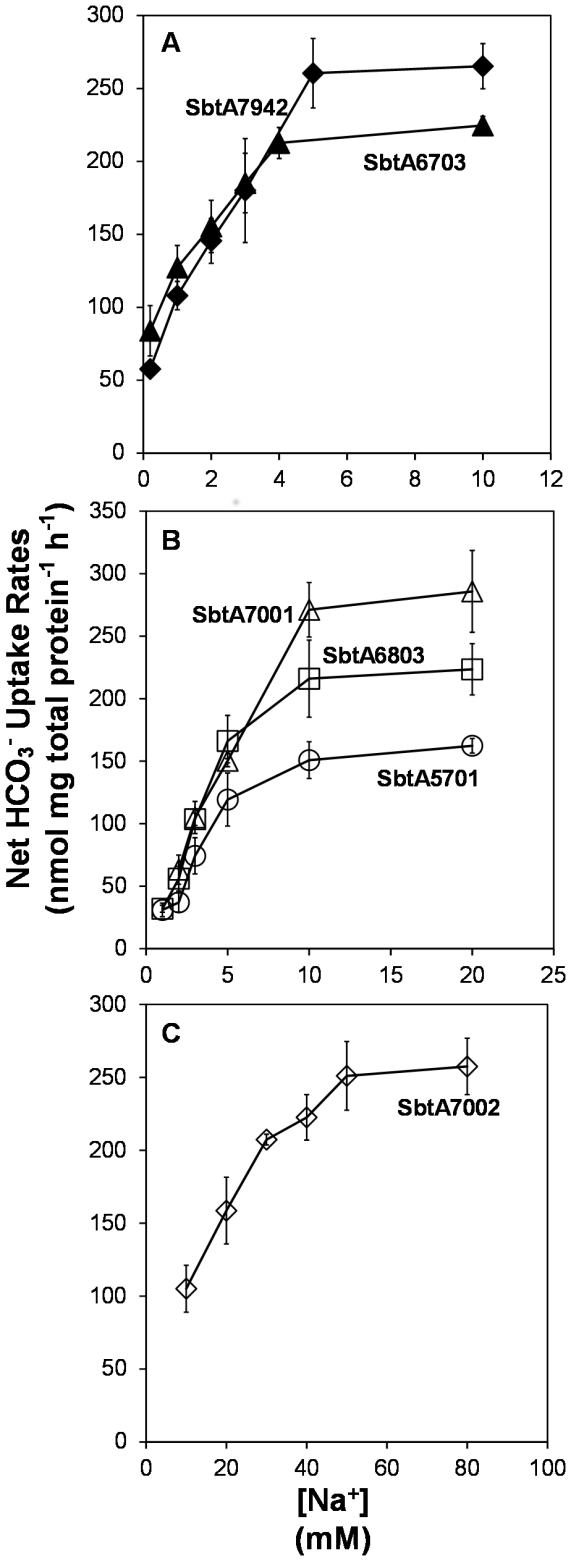
Sodium dependency of HCO_3_^-^ uptake due to expression of various SbtA clones. Cells were spun down and washed twice with CO_2_-free uptake buffer (22 mM potassium phosphate buffer, 20 mM pH 8 Bis-Tris-Propane-HCl pH 8). Additional NaCl was added to cells at various concentrations prior to uptake experiments. Net uptake rates were calculated by subtracting data of *pSE2* empty (18–35 nmol mg total protein^−1^ h^−1^) from raw data of each transporter. Values in the figure are means ± SD (n = 6). SbtA7942, black diamond; SbtA6703, black triangle; SbtA6803, white square; SbtA5701, white circle; SbtA7001, white triangle and SbtA7002, white diamond.

SbtA7942 and SbtA6307 required less Na^+^ compared to the others, with 1.5 mM and 0.8 mM Na^+^ for half maximal activities, respectively (Fig. 3 and Table 2). SbtA6803, SbtA5701 and SbtA7001 had intermediate requirements for Na^+^ and needed 3 to 5 mM Na^+^ to achieve half maximal HCO_3_^-^ uptake rates. The SbtA from a coastal marine species, SbtA7002, had the largest Na^+^ requirement of about 15 mM Na^+^ half maximal HCO_3_^-^ uptake rates. To some extent, the Na^+^ requirements for expressed SbtA clones were related to preferred habitat ranges [6] of the source species of each clone, with freshwater strains (PCC7942, PCC6803, PCC6307) and freshwater/estuarine strains (WH5701, PCC7001) having lower half-requirements than the marine/euryhaline strain, *Synechococcus* PCC7002. All SbtA transporters were saturated by 50 mM NaCl.

**Table 2:** Sodium concentration required for half maximal and maximal activities of SbtA H^14^CO_3_^-^ uptake activity.

SbtA clone	Na^+^ requirement for half maximal activity (mM)	Na^+^ requirement for maximal activity (mM)
SbtA7942	1.5	5
SbtA6803	3	10
SbtA7002	15	50
SbtA6307	0.8	5
SbtA5701	3.5	10
SbtA7001	5	10

Values were derived from the curves in Fig. 3.

### Affinity estimations and maximal HCO_3_^-^ uptake rates of SbtAs

**Figure 4:**
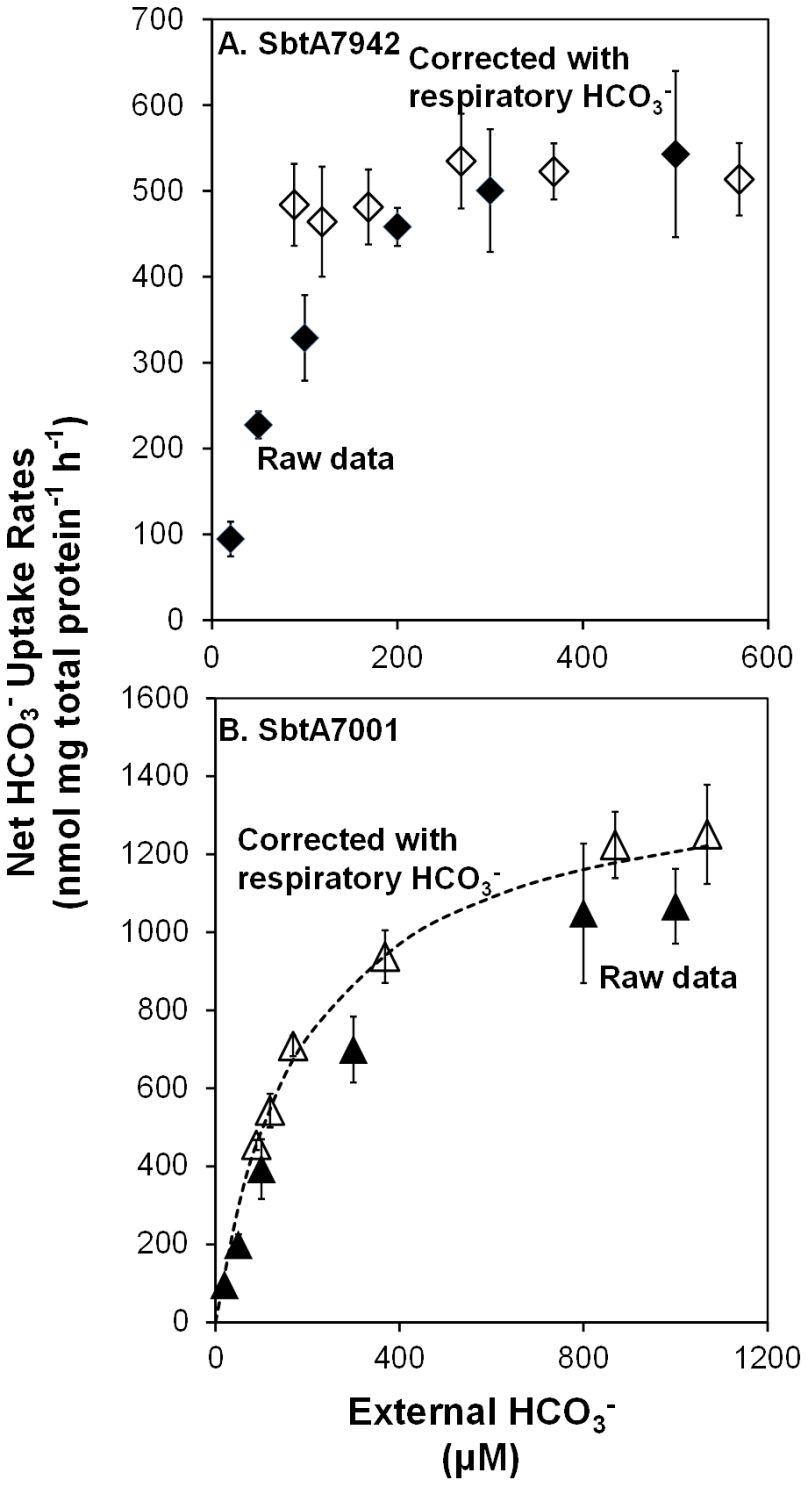
HCO_3_^-^ uptake of SbtA7942 and SbtA7001 against changes at external HCO_3_^-^ levels. Uptake was measured as described in Materials and Methods. Respiratory HCO_3_^-^ levels were measured with MIMS allowing a correction for dilution of ^14^C-HCO_3_^-^ specific activity. Values in the figures are means ± SD (n = 6). A. HCO_3_^-^ uptake of SbtA7942. SbtA7942 Corrected (white diamond) uptake rates were calculated by subtraction of respiratory carbon from the SbtA7942 Raw data (black diamond). Six concentrations of Ci (20 to 500 µM) were injected to *pSE2* or SbtA7942 cells. Raw uptake rates for *pSE2* control were 6 to 199 nmol mg^−1^ h^−1^ and were corrected to 98 to 609 nmol mg^−1^ h^−1^. B. HCO_3_^-^ uptake of SbtA7001. SbtA7001 Corrected uptake rates (white triangle) were calculated by subtraction of respiratory Ci from the SbtA7001 Raw data (black triangle). Six concentrations of Ci (20 to 1000 µM) were injected to *pSE2* or SbtA7001 cells. Raw uptake rates for *pSE2* control were 9 to 294 nmol mg^−1^ h^−1^ and were corrected to 30 to 383 nmol mg^−1^ h^−1^. The theoretical Michaelis-Menten curve (Broken line) was calculated from SbtA7001 Corrected data (R^2^ = 0.9031).

Accurate determination of K_m_[HCO_3_^-^] of high affinity SbtA transporters was difficult in *E. coli* because of CO_2_ generated from cell respiration which altered the effective unlabelled HCO_3_^-^ concentration. Despite all precautions (see material and Methods), around 80 µM Ci was generated due to cell respiration, as determined by mass spectrometer analysis. Most of the respiratory CO_2_ is present as HCO_3_^-^ in the buffer at alkaline pH. This source of HCO_3_^-^ increases the concentration of total HCO_3_^-^ and consequently reduces ^14^C specific activities (CPM nmol^-1^). Taking this into account resulted in the transformation of an initial raw Michaelis-Menten-like curve into a roughly flat line for SbtA7942 (Fig. 4A). This suggested that even the lowest Ci concentration was well above the true K_m_[HCO_3_^-^]. Unfortunately, this was case for all the other SbtA transporters except for SbtA7001. It can only be concluded that the K_m_[HCO_3_^-^] of SbtA7942, SbtA6803, SbtA7002, SbtA6307 and SbtA5701 were under 100 µM (20 µM injected HCO_3_^-^ plus ∼80 µM respiratory HCO_3_^-^). SbtA7001, however, appears to have lower affinity and its K_m_[HCO_3_^-^] was calculated to be 189 µM (Fig. 4B).

Maximal uptake rates for HCO_3_^-^ of the different SbtAs can still be readily determined when respiratory CO_2_ was taken into account (Table 3). SbtA7001 showed a maximal uptake rate for HCO_3_^-^ at over 1200 nmol mg total protein^−1^ h^−1^. SbtA5701 and SbtA6307 had the lowest maximal activity and were able to transport HCO_3_^-^ at 200 nmol and 400 nmol mg total protein^−1^ h^−1^, respectively. SbtA7942, SbtA6803 and SbtA7002 had intermediate maximal HCO_3_^-^ uptake rates, ranging from 500 to 800 nmol mg total protein^−1^ h^−1^. For the BicA bicarbonate transporter a correlation between V_max_ and K_m_[HCO_3_^-^] has been observed for three BicA forms [11], and certainly SbtA7001 has the highest V_max_ and K_m_, but the lack of precise K_m_ data for the other SbtA forms makes full analysis premature at this point in time.

**Table 3:** Kinetics for HCO_3_^-^ uptake properties of SbtA homologs expressed in *E. coli*.

SbtA clone	Km[HCO_3_^-^] (µM)	Maximal HCO_3_^-^ uptake rates (nmol mg total protein^−1^ h^−1^)	Corrected HCO_3_^-^ uptake rates based on relative protein abundance
SbtA7942	<100	559±47	559
SbtA6803	<100	756±14	1844
SbtA7002	<100	519±50	1573
SbtA6307	<100	390±56	1000
SbtA5701	<100	218±19	1816
SbtA7001	189	1238±127	4585

**Figure 5:**
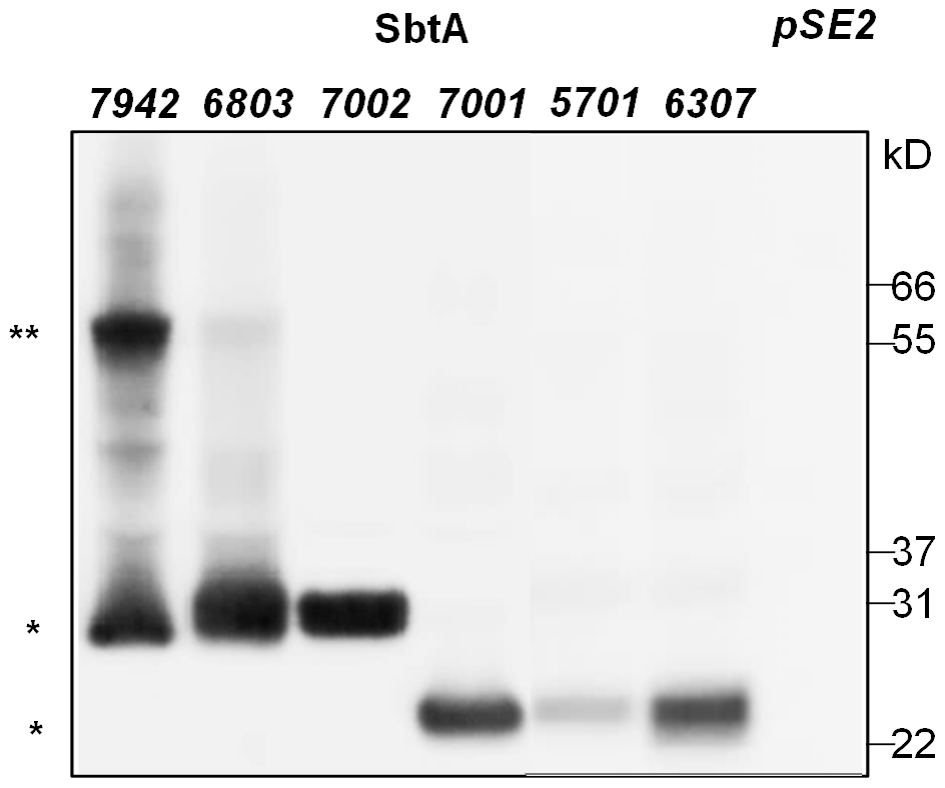
Relative accumulation of SbtA proteins expressed in enriched *E. coli* membrane fractions by western blotting. The respective *sbtA* genes were introduced on *pSE2* plasmids under control of the IPTG inducible *lac* promoter. An empty vector (*pSE2*) served as negative control (right most lane). Gene expression was induced for 2.5 h with 1 mM IPTG. The membrane-enriched protein fractions were isolated, and 50 µg total protein per lane was separated by SDS-PAGE; bands detected by western blotting using the SbtA antibody. *  =  SbtA monomer; **  =  possible dimer of SbtA.

Data were corrected with respiratory Ci. Maximal HCO_3_^-^ uptake rates were calculated from maximal Ci uptake, assuming that 98.1% Ci was HCO_3_^-^ at pH 8. Data were presented as mean ± SD (n = 6). Details of relative abundance of SbtA homologs were included in the Results section. Estimated relative abundances were based on Fig. 5: SbtA7942 (100%), SbtA6803 (41%), SbtA7002 (33%), SbtA 7001 (27%), SbtA5701 (12%) and SbtA6307 (39%).

### Relative abundance of SbtAs in *E. coli*

In order to compare relative expression levels of the different SbtA proteins in *E. coli*, immunodetection was used to investigate abundance of all SbtA proteins in membrane-enriched fractions. A polyclonal anti-SbtA antibody cross-reacting very specifically with all SbtA proteins was used in this study. A single band at a molecular mass roughly 8–10 kD lower than predicted was observed for SbtA7002, SbtA7001, SbtA5701 and SbtA6307 (Fig. 5). Note that aberrant molecular mass on SDS PAGE is a very common observation with highly hydrophobic membrane proteins [17], [23], [24]. Both SbtA7942 and SbtA6803 also showed an additional band at around double the expected molecular weight. This may be a dimeric form of SbtA that has not been entirely disrupted by ionic detergent and reducing reagents. As equal amounts of total protein were loaded on the gel, the relative amount of each SbtA protein could be estimated based on image pixel volumes relative to a dilution series of SbtA7942 ranging from 20 to100% total protein loaded. Estimated relative abundances were: SbtA6803 (41%), SbtA7002 (33%), SbtA 7001 (27%), SbtA5701 (12%) and SbtA6307 (39%).

Determination of relative HCO_3_^-^ uptake rates by correcting for the relative abundances of the various SbtA proteins allows estimation of the specific activity of each SbtA (Table 3). This suggests that there could be up to an eight-fold difference in Vmax, with SbtA7001 having the highest maximal HCO_3_^-^ uptake activity (4585 nmol mg total protein^−1^ h^−1^) and SbtA7942 the lowest (559 nmol mg total protein^−1^ h^−1^).

### Effects of active SbtA on internal Ci pools

It is envisaged that expression of an active HCO_3_^-^ transporter in the chloroplasts of crop plants will need to elevate internal Ci levels [8] to subsequently improve photosynthetic rates. Therefore, we investigated the effects of expression of SbtA transporters on internal Ci pools of *E. coli.* The internal Ci pool (mM) was calculated based on corrected Ci uptake and using cell volumes determined as described in the Methods. Uptake of Ci was measured at 1000 µM injected Ci for SbtA7001 and at 500 µM for the other SbtA transporters to ensure maximal uptake rates for each SbtA transporter were achieved. Data were then corrected for respiratory CO_2_.

**Figure 6:**
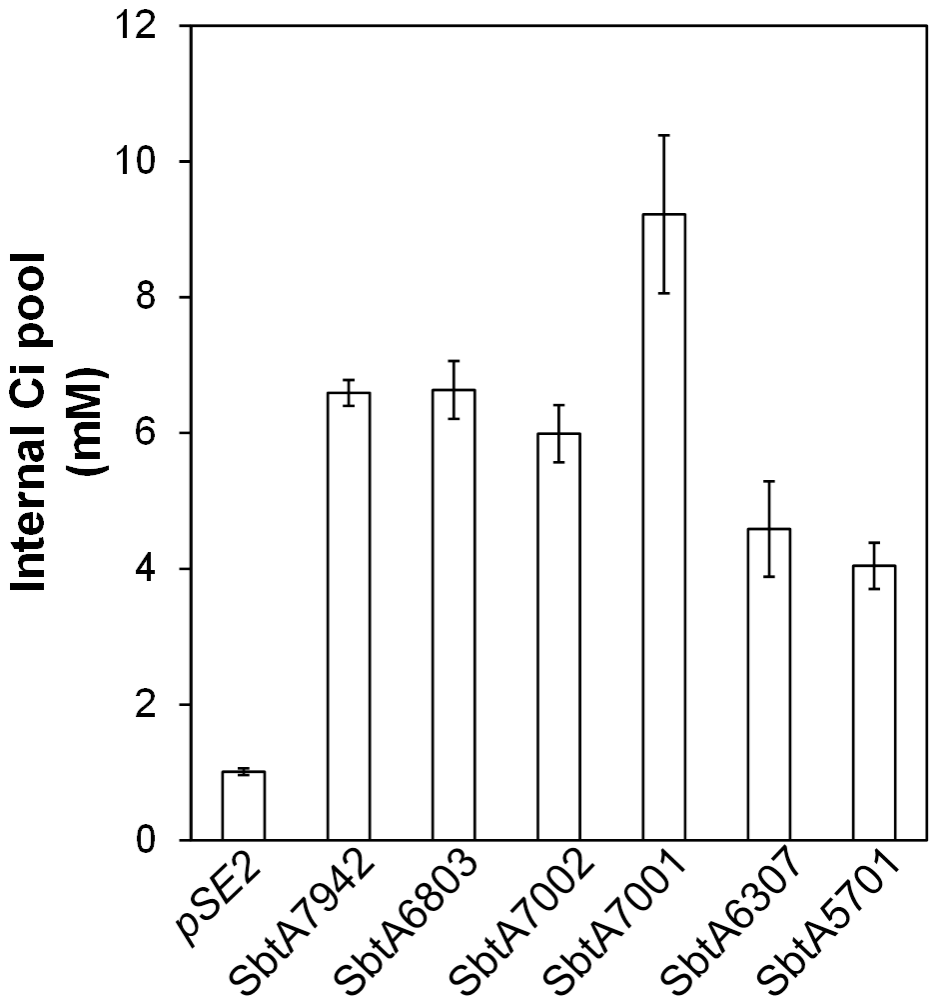
Internal Ci pool sizes of *E. coli* cells with the empty vector or various *sbtA* clones. The expression vector used was the *pSE2* vector. Internal Ci pools (mM) were calculated from maximum Ci uptake and the cell volume of each strain. Ci uptakes were measured in the presence of 500 µM injected H^14^CO_3_^-^ (except SbtA7001 which was 1000 µM) and corrected for respiratory Ci. Data as means ± SD (n = 6). The pools size of cells with each SbtA transporter was significantly different from the pool size of cells with the empty *pSE2* vector as determined with the Welch's T-test (all *p*<0.02).

Internal Ci pool sizes were significantly increased in the presence of active SbtA transporters (Fig. 6). Expression of SbtA7001 led to the most significant increase, of about 8-fold increase compared to *pSE2* only control, while the presence of SbtA5701 resulted in the smallest increase of only 2-fold. This equates to an increase in the internal Ci pool by more than 8 mM for *E. coli* expressing SbtA7001 and by 3–6 mM for the other SbtA transporters relative to controls without expressed transporters.

### The role of SbtB in the regulation of SbtA uptake activity

**Figure 7:**
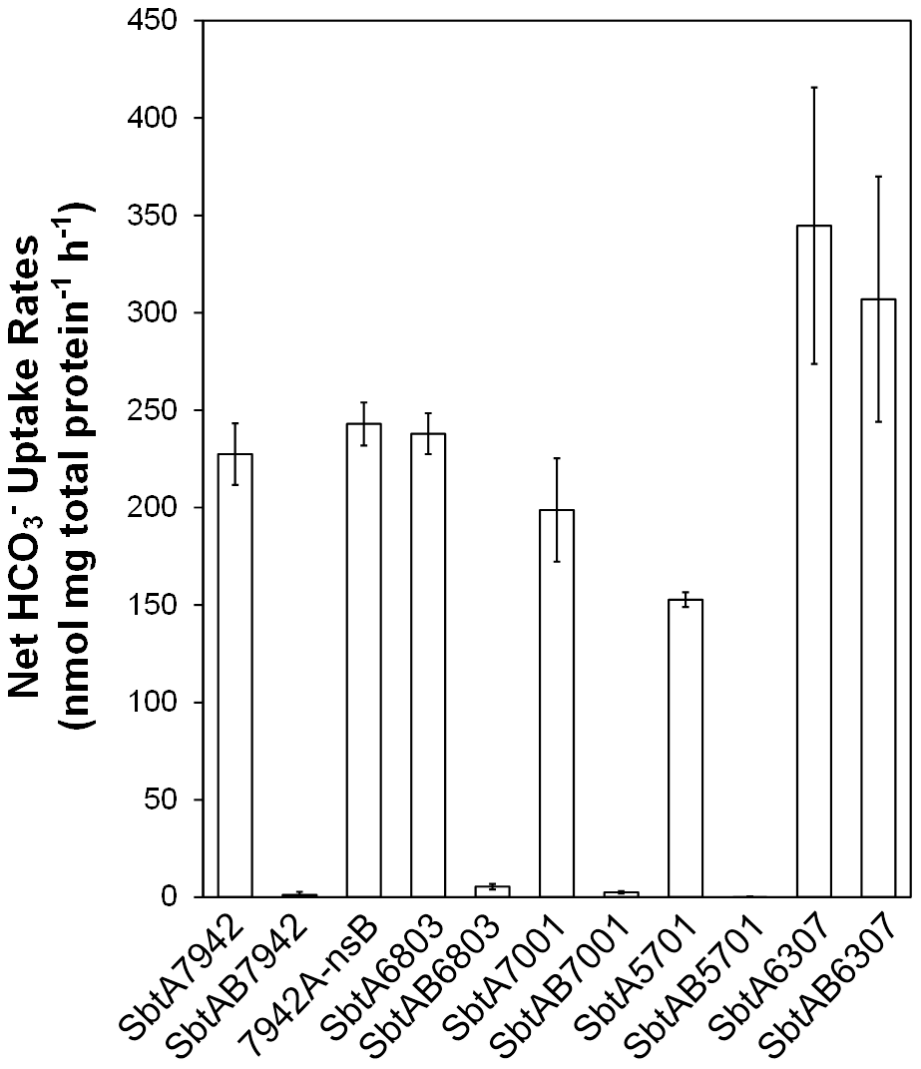
HCO_3_^-^ uptake capacity assessed for five separate SbtAB pairs and 7942A-nsB. Uptake rates were calculated by subtracting data for the empty *pSE2* control (∼22 nmol mg^−1^ h^−1^) from raw data of each strain. Data were not corrected with respiratory Ci as this is encompassed in the control value. Values in the figure are means ± SD (n = 6). The statistical significance of data was analysed with the Welch's T-test. The HCO_3_^-^ uptake rates of SbtA7942, SbtA6803, SbtA7001, SbtA7002 and SbtA5701 were significantly different with or without corresponding SbtB (all *p*<0.01). The HCO_3_^-^ uptake rates of SbtA6307 had no significant difference with or without SbtB6307 (*p* = 0.37). 7942A-nsB showed no significant difference in HCO_3_^-^ uptake rates to SbtA7942 (*p* = 0.59).

The role of SbtB has not yet been determined but the co-location of the *sbtB* gene in, or near, the same operon as the *sbtA* gene suggests a potential role as a regulator of SbtA uptake activity or transcriptional expression. To investigate effects of SbtB on uptake activity of SbtA, five dicistronic *sbtAB* gene pairs were co-expressed from the *lac* promoter in *E. coli* (Fig. 1). All plasmid constructs lacked endogenous promoters for *sbtA* and/or *sbtB* genes to rule out the possibility of transcriptional control of SbtB on *sbtA* transcription. These five pairs were from cyanobacterial strains *Synechococcus elongatus* PCC7942 (SbtAB7942), *Synechocystis sp.* PCC6803 (SbtAB6803), *Cyanobium sp.* PCC7001 (SbtAB7001), *Synechococcus sp.* WH5701 (SbtAB5701) and *Cyanobium gracile* PCC6307 (SbtAB6307). Interestingly, active HCO_3_^-^ uptake was eliminated when SbtA was co-expressed with SbtB for SbtAB7942, SbtAB6803, SbtAB7001 and SbtAB5701 (Fig. 7). This suggests that SbtB may act as an inhibitor of SbtA activity, potentially by binding to SbtA. SbtAB6307 was an exception, with no effect of SbtB on SbtA activity. To date, the reason for the lack of effect is unclear and needs to be investigated further to determine whether SbtB has a different role in this species or there is a problem with the expression of SbtB. When SbtB was not present the transporters all showed normal uptake of HCO_3_^-^ (Fig. 7).

We investigated the possible regulatory role of SbtB further for SbtAB7942. Firstly, we generated a construct in which the start codon of SbtB was mutated from ATG to GGG, in construct 7942A-nsB (Fig. 1). This was designed to abolish translation of SbtB without impacting on translation of SbtA. This construct showed the same SbtA activity as the construct lacking SbtB (Fig. 7), indicating that inhibition is dependent on the presence of the SbtB protein.

Secondly, we investigated whether SbtA and SbtB interact. SbtA and SbtB were tagged with c-Myc and HA-His6, respectively, to allow immunochemical detection and affinity purification of SbtB (Table 4 and Fig. 1). The preliminary immunochemical detection of SbtA and SbtB with Western blotting showed that when both proteins were present, SbtA and SbtB were detected in the membrane fraction (S2 Fig.). However, when SbtB was expressed alone, it was detected in the soluble protein fraction (S2 Fig.). This result suggested a physical interaction between the two proteins.

**Table 4:** List of constructs involved in characterisation of SbtA transporters.

*pSE2* Construct names		Description
**SbtA constructs**	SbtA7942	*sbtA* from *Synechococcus* sp. PCC7942
	SbtA6803	*sbtA* from *Synechocystis* sp. PCC6803
	SbtA7001	*sbtA* from *Cyanobium* sp. PCC7001
	SbtA7002	*sbtA* from *Synechococcus* sp. PCC 7002
	SbtA6307	*sbtA* from *Cyanobium* sp. PCC6307
	SbtA5701	*sbtA* from *Synechococcus* sp. WH5701
**SbtAB constructs**	SbtAB7942	Artificial dicistronic clone for *sbtA* and *sbtB*, *Synechococcus* PCC7942
	SbtAB6803	*sbtA and sbtB6803* cloned as a natural dicistronic context
	SbtAB7001	*sbtA and sbtB7001* cloned as a natural dicistronic context
	SbtAB6307	*sbtA and sbtB6307* cloned as a natural dicistronic context
	SbtAB5701	*sbtA and sbtB5701* cloned as a natural dicistronic context
	7942A-nsB	Start codon of *sbtB* altered (ATG to GGG) in SbtAB7942
**Constructs for protein-protein interactions**	7942AMyc	c-Myc tag fused into loop 5/6 of *sbtA7942* in SbtA7942 construct (at E203)
	7942AB-HAH6	HA tag fused at the C-terminus of *sbtB*, based on SbtAB7942 construct
	7942B-HAH6	*sbtB* only generated from ABHAH67942 by PCR
	7942AMBH	c-Myc tag fused into loop 5/6 of *sbtA7942* based on the ABHAH6-7942 construct

All constructs were based on pSE2 vector in which the expression of proteins was driven by the *lac* promoter. A c-Myc tag was fused to the 5/6 loop of SbtA7942 after position E203 of SbtA. The HA and His6 tags were fused to the C-terminus of SbtB7942. A schematic illustration of the constructs can be found in Fig. 1.

**Figure 8:**
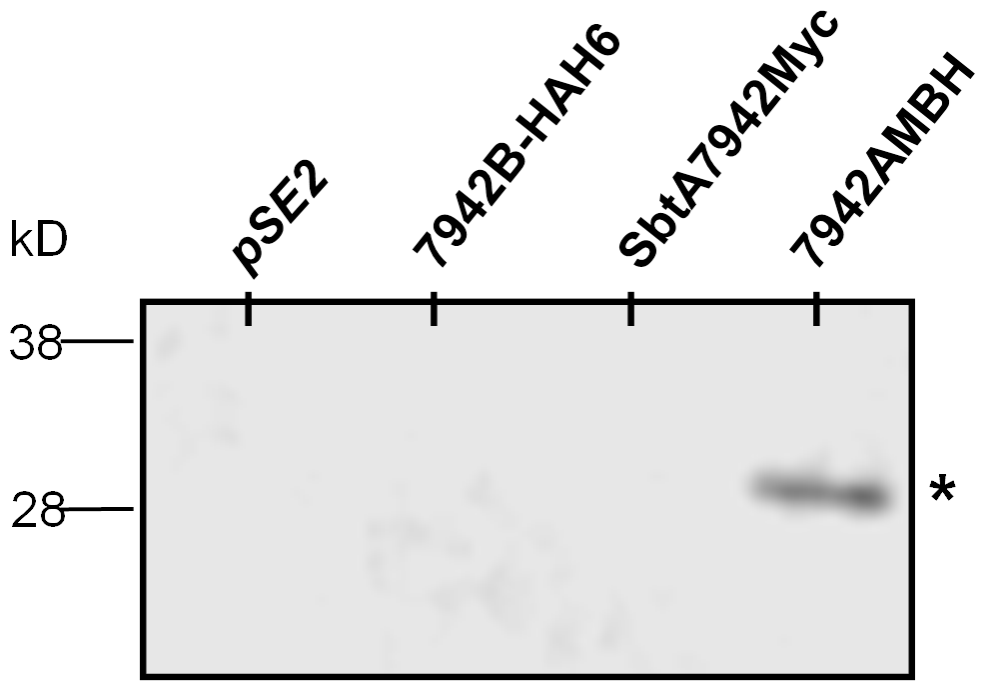
Isolation of SbtA7942 by IMAC using SbtB-HAH6 as binding partner and detected by western blotting. Gene expression was induced for 2.5 h with 1 mM IPTG. The membrane-enriched protein fractions of *E. coli* containing the empty *pSE2*, 7942AMyc and 7942AMBH and 7942B-HAH6 vector were used for IMAC isolation. A total of 40 µg total protein of IMAC elutes per lane was separated by SDS-PAGE and subjected to Western blotting. Proteins were detected with the Anti-c-Myc antibody.

To confirm an interaction between SbtA and SbtB, we tested whether the two proteins co-purify from solubilised membrane-enriched fractions. Affinity chromatography was used to isolate His-tagged SbtB from the enriched membrane fraction in a construct expressing both SbtA and SbtB (7942AmBH, Table 4). Since only SbtB was tagged with His6, SbtA should not be detected unless it interacts with SbtB. Western blotting with the anti-c-Myc antibody showed that SbtA could be detected after purification (Fig. 8). No SbtA was detected when the affinity chromatography was repeated with the same construct lacking either SbtA or SbtB (Fig. 8). This strongly suggests that SbtA is purified because it interacts with SbtB, rather than through a non-specific interaction with the resin of the chromatography column.

## Discussion

### Characterisation of SbtA homologs in *E. coli*

In this study, we successfully demonstrated functional expression of a number of HCO_3_^-^ transporters and their homologs in *E. coli*. To our knowledge, this is first time this has been achieved in *E. coli* as a heterologous expression system. Six cyanobacterial SbtA homologs were shown to display HCO_3_^-^ uptake while members of the BicA and BCT1 families lacked any detectable uptake. It seems that at least for BicA7002, additional regulator(s) are required for its function in *E. coli*, because we were able to detect BicA in the enriched membrane fraction of *E. coli* (S3 Fig.). The experiments to detect BCT1 proteins were not conducted due to the lack of antibodies. In addition, this is the first experimental evidence that SbtA7942, SbtA7001, SbtA6307 and SbtA5701 homologs function as HCO_3_^-^ transporters. The latter three homologs were derived from transitional α-cyanobacteria of the *Cyanobium* clade but their *sbtA* genes are thought to have originated from β-cyanobacteria [13]. The other three homologs were derived from β-cyanobacteria. All six SbtA homologs were able to complement the EDCM636 CA-deficient mutant to restore growth at atmospheric CO_2_ levels (Fig. 2).

The six SbtA homologs chosen from β-cyanobacteria and transitional strains represent two groups showing minor protein sequence differences. Intriguingly, the most notable variation is in the size of the loop between helices 5 and 6 which separates the two homologous halves of the transporter [9], [13]. The loop is consistently 35–40 amino acids shorter in the SbtA proteins from transitional strains. We were interested in whether this correlated with any functional differences in Na^+^ requirements, maximal HCO_3_^-^ uptake rates or K_m_[HCO_3_^-^]. However, the six SbtA homologs showed various Na^+^ requirements and HCO_3_^-^ uptake kinetics, unrelated to the sizes of the loop between helix 5 and 6, suggesting that the determinants of the properties we examined lie in other areas of difference.

### Advantages and limitations of an *E. coli* system for analysis of cyanobacterial HCO_3_^-^ transporters

Our main objective was to study cyanobacterial HCO_3_^-^ transporters in a heterologous background where analysis was unlikely to be compromised by cyanobacterial regulatory factors involved in their activation/deactivation. Given its standard use in the laboratory for recombinant protein expression and molecular genetics as well as availability of a wide range of metabolic mutants, including carbonic anhydrase, *E. coli* was our system of choice. We developed two independent assays for function: a silicon oil centrifugation-filtration uptake assay and a complementation assay in *E. coli*. We were able to show that at least six members of the SbtA HCO_3_^-^ transporter family are expressed and functional in the absence of other cyanobacterial components and active photosynthesis. In other words, SbtA alone has the desirable property of being constitutively active. However, both assays have limitations that need to be taken into consideration. While the uptake assay allowed us to identify some kinetic parameters of the transporters, a drawback of this system is that we were not able to completely remove inorganic carbon, mainly CO_2_, generated by cell respiration. In contrast, respiratory Ci can be conveniently removed by a short period of photosynthetic CO_2_ fixation in a closed cuvette when using photosynthetic organisms for this type of analysis. In *E. coli*, the presence of respiratory Ci leads to dilution of radioactivity and an inability to provide cells with near-zero levels of Ci during uptake assays. The residual Ci concentration can be measured and corrected for, however, it remains impossible to measure uptake at Ci concentrations below the residual level. This is not problematic for kinetic measurements for HCO_3_^-^ transporters with medium to low affinity, as illustrated by the case of SbtA7001. However, existence of residual Ci hinders accurate determination of K_m_[HCO_3_^-^] for high affinity HCO_3_^-^ transporters, as illustrated by the case of the remaining SbtA transporters. For example, in cyanobacteria, the SbtA7002 form is estimated to have a transport affinity as low as 2 µM [11].

Complementation of the CA-deficient *E. coli* strain, EDCM636, provides a convenient screen for function of individual transporters (Fig. 2). However, the strain reverts to wild type at a relatively high frequency as a consequence of the presence of a second wild type CA gene, *cynT*, that is not normally expressed, leading to selection for expression in any screening assay. In fact, about 12% of plated EDCM636 colonies regained CA activity and lost the need for high CO_2_ for growth [21]. In our case, we found that the occurrence of reversion could be reduced by taking extra precautions, for example using fresh cells from glycerol stocks. Nevertheless, this strategy would be unsuitable for large scale functional screening of HCO_3_^-^ transporters, for example, using cDNA or mutant libraries. In spite of these drawbacks, the two assays described here have been valuable in identifying and analysing HCO_3_^-^ transporters in a heterologous, non-photosynthetic system and will be useful for future investigations of SbtA structure and function.

### Post-translational regulation of SbtA by SbtB

One novel and important finding of this study is that SbtB serves as a post-translational regulator of SbtA. Firstly, co-expression of SbtB inhibited HCO_3_^-^ uptake by SbtA in four out of five *sbtAB* expression pairs. The only exception was SbtAB6307 in which the uptake activity of SbtA6307 was not affected for unknown reasons, which could be as simple as lack of expression of the SbtB6307 protein. There are no SbtB antibodies available for testing this possibility. Generation of a tagged version of SbtB6307 with a HA or c-Myc epitope detectable by commercially available antibodies would be required, which is part of future investigations.

Secondly, the requirement for synthesis of the SbtB protein for inhibition and the fact that substantial amounts of SbtA protein accumulate in the presence of SbtB rules out regulation of expression at the transcriptional or translational level. Thirdly, there is a strong indication for direct protein-protein interaction between SbtA and SbtB. SbtA and SbtB was co-purified using a polyhistidine tag located on SbtB, indicating a strong physical interaction between SbtA and SbtB. In addition, immunodetection showed that SbtB7942 was only detectable in the plasma membrane when co-expressed with SbtA in *E. coli* (S2 Fig.). It is likely that in *E. coli*, SbtB regulates SbtA independently of secondary regulation processes in cyanobacteria. As such, it is interesting to speculate that in cyanobacteria SbtB might acts as a “curfew” protein to help inactivate SbtA in the dark, and that cyanobacteria would also have a mechanism to “unlock” SbtA in the light. SbtB shares low similarity (21% identity) in amino acid sequence with cyanobacterial P_II_ proteins, and an unpublished crystal structure for SbtB from *Anabaena* (www.ncbi.nlm.nih.gov structure 3DFE) shows that β-SbtB has a very similar fold to P_II_ (GlnB; structure 1QY7) from cyanobacteria. P_II_/GlnB proteins form trimers, are widely distributed in many bacteria, and are key regulators of nitrogen metabolism. This occurs through binding of effector molecules, indicating nitrogen status such as oxo-glutarate and ADP and post-translational interactions with a range of proteins [25].

It is noteworthy that the trimeric AmtB ammonia channel from *E. coli* is regulated by the binding of the GlnK trimer (P_II_ homolog), with AmtB being inactive for ammonia influx when GlnK is bound, and active when unbound, at high levels of oxo-glutarate, ATP and Mg^2+^ [26], [27]. AmtB-GlnK could therefore make a useful working model for analysis of SbtA-SbtB regulation, despite potential differences in effectors required. Furthermore, because SbtB crystallises as a trimer (see above), it seems sensible to postulate that SbtA functions as a trimer; data suggesting that SbtA6803 runs on native gels as a 160 kDa tetramer [15] could also potentially be re-interpreted as 156 kDa expected size (3 times 40 kDa for SbtA plus 3 times 12 kDa SbtB). Future investigation is required to better understand the mechanism of SbtA regulation by SbtB, its role in modulating HCO_3_^-^ uptake activity of SbtA, and whether SbtB could also be involved in other signalling pathways in a similar way to P_II_ (GlnB).

### SbtA candidates for expression in crop plants

One longer term goal of our research is to identify candidate HCO_3_^-^ transporters to be expressed in crops [7], [8]. This requires that the transporters are active in heterologous systems and have kinetic properties that are consistent with functional expression in the chloroplast. Several SbtA homologs we tested are good candidates, with the best able to increase the Ci pool inside *E. coli* cells by up to 9 mM. We tested HCO_3_^-^ transporters from the BicA homolog grouping and found that none was functional in *E. coli* under our experimental conditions. Whether this is due to a need for unidentified regulatory factors is not yet known. However, all members of the SbtA family were functional and also showed interesting variation in their kinetic characteristics, allowing selection for those with the most potential for chloroplast expression.

It is estimated that at least 250 µM HCO_3_^-^ is present in the C3 leaf cytosol under ambient air [28] and that 1 to 3 mM Na^+^ is present in the cytoplasm [29], as an inwardly directed Na^+^ gradient across the chloroplast inner membrane [30]. This could potentially provide a suitable environment for increased accumulation of Ci in the chloroplast due to expression of at least some of the SbtA homologs characterised here. The K_m_[HCO_3_^-^] of all SbtAs tested was below the 250 µM HCO_3_^-^ present in the leaf cytosol. SbtA7942, SbtA6803, SbtA6307 and SbtA5701 may represent more suitable candidates to be expressed in crops because of their lower requirements for Na^+^, with SbtA7942 and SbtA6307 needing only 1.5 mM and 0.8 mM Na^+^ respectively for half maximal uptake (Table 2). We are currently investigating the suitability of SbtA for functional expression in C3 chloroplasts.

## Materials and Methods

### Bacterial strains and growth conditions

*E. coli* K12 strain DH5α (*F– Φ80lacZΔM15 Δ(lacZYA-argF) U169 recA1 endA1 hsdR17 (rK–, mK+) phoA supE44 λ– thi-1 gyrA96 relA1*) was used routinely for cloning, storage of plasmids and general expression of membrane proteins. *E. coli* for screening of HCO_3_^-^ transporters was a CA-deficient strain EDCM636, which is derived from *E. coli* MG1655 (*F- λ-ilvG-rfb-50 rph-1*) harbouring a kanamycin resistance marker replacing a deletion of the CA encoding gene *can* (*Δcan*) [21]. EDCM636a, a strain with restored CA function, was specially selected to provide a positive control in dilution spotting assays (see below). A second control strain also contained an empty *pSE2* vector. Genes encoding for membrane proteins were cloned into the *pSE2* vector where their expression was driven by the IPTG-inducible *lacZ* promoter [31]. The *pSE2* vector carries a spectinomycin resistance gene as selectable marker. The main plasmid constructs involved in the characterisation of SbtA transporters are listed in Table 4 and Fig. 1.

Luria–Bertani (LB) broth and LB agar were used for routine bacterial growth in liquid culture while shaking or on solid medium, respectively. Unless specified, cells were grown at 37°C. For dilution spotting assay, *E. coli* was cultured on LB agar or M9 minimal agar with 0.4% glycerol [32]. Where applicable, antibiotics were added to the following final concentrations: kanamycin at 50 µg ml^−1^ and spectinomycin at 100 µg ml^−1^.

### Dilution spotting assay

*E. coli* strains were grown on LB agar plates overnight. For strain EDCM636, 0.1 mM sodium azide was added to plates to induce expression of *cynT* [21]. The next morning, cells were resuspended in MilliQ water to OD600 of 0.1, and then diluted to 10^−3^, 10^−4^ and 10^−5^. An aliquot of 10 µl of each dilution was pipetted onto LB agar containing 20 mM Epps-HCl pH 8 or M9 agar with 0.4% glycerol and 20 mM Epps-HCl pH 8 supplemented with the appropriate antibiotics. Protein expression was induced with IPTG at a final concentration of 0.2 mM. Plates were incubated at 24°C for 2 days (LB) and 6 days (M9).

### Bicarbonate uptake measurements

Bacterial strains for HCO_3_^-^ transporter expression and functional analysis were pre-grown for 16 h in 3 ml LB broth with spectinomycin, inoculated into 10 ml LB-spectinomycin broth and grown for 1 h. A final concentration of 1 mM IPTG was added to induce transporter gene expression for 3 h unless stated otherwise. Optimisation experiments showed the level of expression increased for 4 h IPTG induction but declined subsequently (S4 Fig.). Cells were harvested by centrifugation at 9,000 *g* for 30 s and washed twice with CO_2_-free uptake buffer (22 mM potassium phosphate, 20 mM Bis-Tris-Propane-HCl pH 8 and 50 mM NaCl. Modified uptake buffers with varying concentrations of Na^+^ were used in experiments to determine Na^+^ dependency of HCO_3_^-^ uptake. To remove CO_2_, the buffer was bubbled with high purity N_2_ for 3 days. Immediately before each uptake assay, cell aliquots were spun down and resuspended in CO_2_-free uptake buffer to minimize the time for respiratory CO_2_ release into the buffer.

Inorganic carbon uptake was determined by the silicon oil centrifugation-filtration assay described previously [33]. A stock solution of radioactive NaH^14^CO_3_ in “cold” NaHCO_3_ (25 mM, 0.11 mCi ml^−1^ pH 9.5) was added to cells at a final concentration of 50 µM (additions of NaH^14^CO_3_ were varied for kinetic measurements), cells were mixed and 100 µl was aliquots were transferred to micro-centrifuge tubes containing 5 µl of “kill” solution (3 M NaOH, 50% methanol) overlaid with 50 µl silicon oil mixture (AR20:AR200 4∶3.5 v/v). Bicarbonate uptake was stopped after 30 s by centrifugation, which was the shortest time in which HCO_3_^-^ uptake reached saturation (S5 Fig.). Tubes were frozen instantaneously in liquid nitrogen for further processing.

The tips of micro-centrifuge tubes containing the cell pellet in “kill” solution were cut off, cell pellets resuspended in 300 µl 2 M NaOH in scintillation vials, and 3 ml scintillation fluid (Ultima Gold XR, PerkinElmer) was added before measuring ^14^C CPM in a Beckman-Coulter scintillation counter. The specific activity of NaH^14^CO_3_ stock solution was calculated from CPM of 1 µl in 200 µl 2 M NaOH. Respiratory CO_2_ contamination was determined from cells treated as for H^14^CO_3_^-^ uptake experiments except using non-radioactive uptake buffer. After cells were spun down the supernatant was immediately transferred to a new tube, stored frozen and total Ci in the supernatant was measured with a membrane inlet mass spectrometer [34]. HCO_3_^-^ uptake rates were calculated as 98.1% of the raw Ci uptake rates based on the pKa of CO_2_ to HCO_3_^-^ at pH 8, 24°C and the ionic strength of the assay buffer [35]. Total protein concentration of each sample was determined using a BCA protein assay kit (Pierce) according to the manufacturer's protocol with bovine serum albumin as a standard.

### Cell volume measurements

Silicon oil centrifugation-filtration removes most excess buffer as cells are spun down through the silicon oil layer except for a thin water (buffer) shell that forms around each cell. To determine the true cell volume, the total of the cell space plus the water shell is estimated from tritiated (^3^H) water which can enter *E. coli* cells and outer space. The water shell is estimated from ^14^C-Inulin, which cannot enter *E. coli* cells [36]. Thus, cell volume can be calculated by subtracting the water shell volume from the total.

Silicon oil centrifugation-filtration assays were performed as described above except that tritium or ^14^C-inulin was added to cells at a final concentration of 0.3 µCi ml^−1^. The incubation time was 10 min for tritium and 30 s for ^14^C-inulin. After centrifugation, 1 µl of the supernatant in each tube was kept for determination of specific activities. Cell volume (µl) was calculated for 1 ml cells at OD600 = 1. Cells containing the *pSE2* vector had a combined cell volume of 2 to 2.5 µl whereas cells expressing the SbtA PCC7942 protein had a combined cell volume of 1 to 1.5 µl (averaged from at least 3 biological replications).

### Preparation of membrane-enriched protein fractions of *E. coli*

Cell cultures grown for 14 h in LB broth with spectinomycin were diluted 1∶3, and after 1 h cells were induced for 2.5 h with 1 mM IPTG (final concentration). Cells were washed twice in lysis buffer (100 mM NaCl, 10 mM MgCl_2_ and 25 mM Tris-HCl, pH 8.0). After one freeze and thaw cycle, cell pellets were resuspended in lysis buffer with 1.4% (v/v) protease inhibitor (PI) cocktail (Complete mini, Roche) and approx. 100 µL of 0.1 mm glass beads (Sigma, USA). Cells were disrupted in a Tissuelyzer (Retsch, Germany) shaking for 5 min at 30 Hz in 1.5 mL microfuge tubes. Cell debris was removed by centrifugation for 15 s at 14,000 *g* at 4°C and transfer of the supernatants to new tubes. Crude membranes were collected by centrifugation at 14,000 *g* at 4°C for 10 min. For immunodetection, the supernatant (soluble protein fraction) and the pellets (crude membrane fraction) were supplemented with sodium dodecyl sulfate (SDS) sample buffer to final concentrations of 62.5 mM Tris-HCl, pH 6.8, 4% (w/v) SDS, 1 mM dithiothreitol (DTT) and 10% glycerol. Both fractions were incubated at 70°C for 20 min. The crude membrane fraction was centrifuged at 14,000 *g* for 15 min to precipitate insolubles. The total protein concentration of soluble protein fraction and enriched membrane fraction was determined with a detergent compatible (DC) protein assay kit (BioRad). Bromophenol blue (2 µg ml^−1^ final) was added prior to analysis by SDS-PAGE.

### Isolation of SbtA:SbtB complexes

For isolation of SbtA:SbtB-HA-H6 complexes a crude membrane fraction was prepared and resuspended in buffer A (50 mM Bis-Tris pH 6.0, 2 mM CaCl_2_, 1 mM DTT, 10% glycerol with PI), frozen in liquid nitrogen and stored at -20°C.

Immobilized metal affinity chromatography (IMAC) was used for protein purification adapted from the method for isolating the native *E. coli* respiratory Complex I [37]. The following steps were carried out at 4°C. In brief, dodecyl-β-D-maltoside (DDM) was added to the crude membrane fraction to a final concentration of 1.2% (w/v). Samples were gently mixed for 1 h and centrifuged in a bench-top micro-centrifuge at 14,000 *g* for 20 min. The supernatant was transferred to a new tube, gradually supplemented with NaCl to a final concentration of 200 mM and mixed with IMAC resin (Profinity IMAC Ni-charged resins, BioRad) equilibrated with buffer A. The mixture was incubated with gentle mixing for 1 h, loaded onto a gravity packed column, and then washed with 2 column volumes of wash buffer (buffer A, 200 mM NaCl, 0.1% DDM and 5 mM histidine). The proteins were eluted with buffer A containing 200 mM NaCl, 0.1% DDM and 200 mM histidine. The eluates were mixed with SDS sample buffer and the concentration of total protein content was determined as described above.

### SDS-PAGE and western blotting

*E. coli* membrane and soluble protein fractions were separated by SDS-PAGE on 4-12% Bis-Tris protein gels (NuPAGE, Invitrogen, USA) as described by the manufacturer. The expression level of SbtA was detected immuno-chemically after transfer to PVDF membrane with a polyclonal antibody (Agrisera, Sweden) directed against a conserved epitope of SbtA proteins from many β-cyanobacteria (PTLRAGIPSANPSAY, S6 Fig.). Tagged proteins were detected with monoclonal antibodies against these epitopes, anti-c-Myc (against EQKLISEEDL) or anti-HA (against YPYDVPDYA) (Sigma, USA). Proteins were visualized by fluorescence detection with an alkaline phosphatase-conjugated secondary antibody and the AttoPhos detection system (Promega, USA) on a Versadoc imager (BioRad, USA). Dilution series of the crude membrane fraction of SbtA7942 were loaded onto SDS-PAGE gels to ensure that the amount of proteins in samples were in the linear range for semi-quantitative analyses using Quantity One software (BioRad, USA).

## Supporting Information

S1 Fig **The effect of KCl and NaCl on the HCO_3_^-^ uptake rates by SbtAs.** Cells were prepared as described in the Methods except that a modified CO_2_-free buffer with 1 mM NaCl was used. Five mM KCl or NaCl was added to cells before the uptake experiments, which resulted in 1 mM NaCl +5 mM KCl or 6 mM NaCl, respectively. Comparable amount of MilliQ water was added to cells as the negative controls (1 mM NaCl + MilliQ). The uptake rates were calculated by subtracting data of the empty *pSE2* vector (25∼30 nmol mg total protein^-1^ hour^-1^) from raw data for each transporter. Values in the figure are means ± SD (n = 6).
(TIF)

S2 Fig **Detection of SbtA7942 and SbtB7942 proteins in** ***E. coli*** **by western blotting.** Gene expression was induced for 2.5 h with 1 mM IPTG. The soluble protein (S) and the membrane-enriched protein (M) fractions of *E. coli* containing the empty *pSE2*, 7942AB-HAH6 and 7942B-HAH6 vectors were used. A total of 30 µg total protein of each fraction per lane was separated by SDS-PAGE and subjected to Western blotting. Proteins were detected with the antibody cocktail of the SbtA antibody and the anti-HA antibody. *  =  SbtA monomer; #  =  SbtB monomer; ##  =  possible dimer of SbtB.
(TIF)

S3 Fig **Detection of BicA7002 protein in the plasma membrane of** ***E. coli*** **by western blotting.** Gene expression was induced for 2.5 h with 1 mM IPTG. The membrane-enriched protein fractions of *E. coli* containing the empty *pSE2* and BicA7002 vectors were used. A total of 30 µg total protein of each fraction per lane was separated by SDS-PAGE and subjected to Western blotting. Proteins were detected with the antibody targeting the STAS domain of BicA. *  =  BicA monomer; **  =  possible dimer of BicA.
(TIF)

S4 Fig **Optimisation of the induction time required for expression of SbtA7942 and SbtA7001.** Cultures were prepared as described in Materials and Methods. Expression of SbtA7942 (diamond) and SbtA7001 (triangle) was induced by adding IPTG (1 mM) for up to 5 hours with samples taken every hour to determine uptake rates. Uptake experiments were performed in the presence of 50 mM NaCl and 50 µM H^14^CO_3_^-^. Net uptake was calculated by subtracting data of *pSE2empty* vector (25∼30 nmol mg total protein^−1^ hour^−1^) from raw data for each transporter. Values in the figure are means ± SD (n = 6).
(TIF)

S5 Fig **Uptake time course for SbtA7942 and SbtA7001.** Cultures were prepared as described in Materials and Methods. Uptake experiments were done in the presence of 50 mM NaCl and 50 µM H^14^CO_3_^-^. Cells were incubated with H^14^CO_3_^-^ for 0.5, 1, 2 and 4 mins. Net uptake was calculated by subtracting data of *pSE2empty* control (0.38∼0.55 nmol mg total protein^−1^) from raw data of SbtA7942 (diamond) and SbtA7001 (triangle). Values in the figure are means ± SD (n = 6).
(TIF)

S6 Fig **An alignment of the six SbtA forms used in the present study.** The clones from β-cyanobacteria were *Synechococcus elongatus sp*. PCC7942 (SynPCC7942; freshwater), *Synechococcus elongatus sp*. PCC7002 (SynPCC7002; coastal/estuarine) and *Synechocystis sp*. PCC6803 (SycPCC6803; freshwater). The clones from α-cyanobacterial transitions strains were from *Cyanobium spp*. PCC6307 (CynPCC6307) and PCC7001 (CynPCC7001) and from *Synechococcus* WH5701 (SynWH5701). The positions of the membrane helices previously determined for *Synechocystis* PCC6803 SbtA are shown in purple. The conserved epitope region used for raising an antibody is shown in red. Residues are shaded according the functional categories: hydrophobic (green), positively charged (red), polar (orange) and aromatic (blue).
(TIF)
