# Supplementary figures and images for: Characterisation of Cyanobacterial Bicarbonate Transporters in E. coli Shows that SbtA Homologs Are Functional in This Heterologous Expression System
Source: PLoS One. 2014 Dec 23;9(12):e115905. doi: 10.1371/journal.pone.0115905 (PMC4275256; doi:10.1371/journal.pone.0115905)

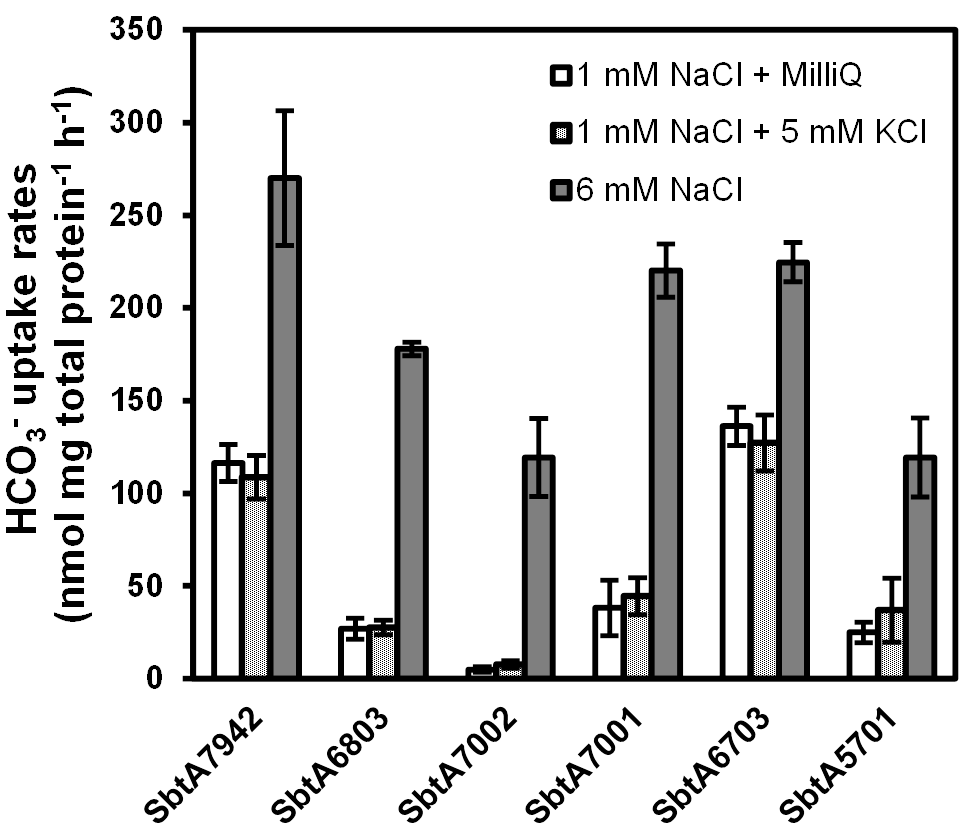

Supplement: S1 Fig — The effect of KCl and NaCl on the HCO3- uptake rates by SbtAs. Cells were prepared as described in the Methods except that a modified CO2-free buffer with 1 mM NaCl was used. Five mM KCl or NaCl was added to cells before the uptake experiments, which resulted in 1 mM NaCl +5 mM KCl or 6 mM NaCl, respectively. Comparable amount of MilliQ water was added to cells as the negative controls (1 mM NaCl + MilliQ). The uptake rates were calculated by subtracting data of the empty pSE2 vector (25∼30 nmol mg total protein-1 hour-1) from raw data for each transporter. Values in the figure are means ± SD (n = 6). (TIF) [file pone.0115905.s001.tif]

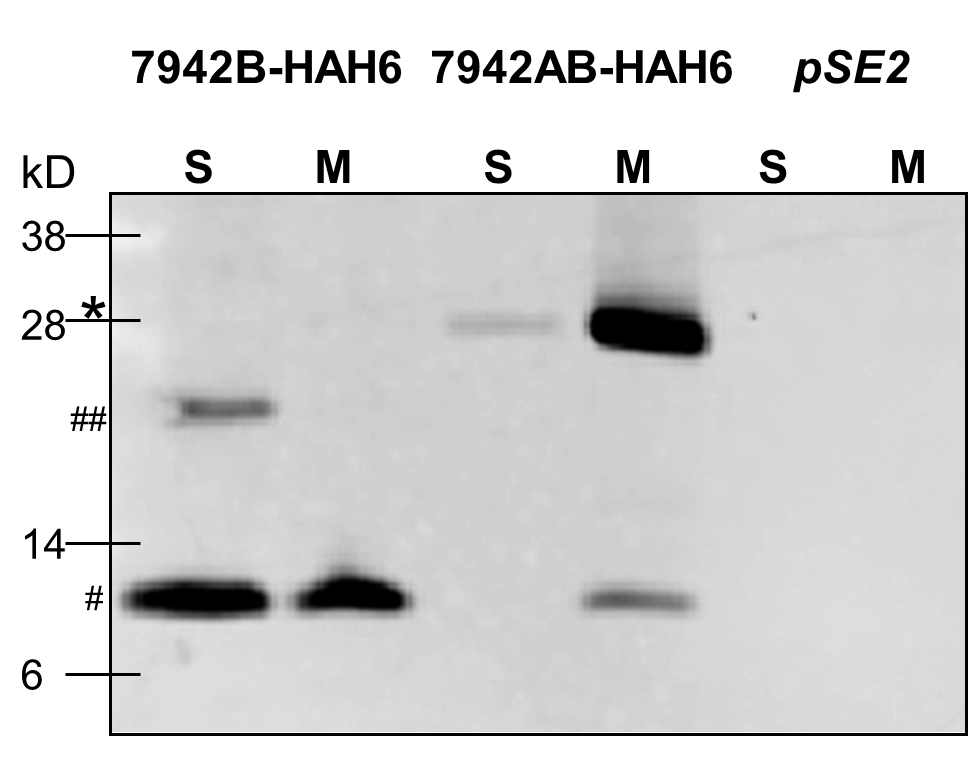

Supplement: S2 Fig — Detection of SbtA7942 and SbtB7942 proteins in E. coli by western blotting. Gene expression was induced for 2.5 h with 1 mM IPTG. The soluble protein (S) and the membrane-enriched protein (M) fractions of E. coli containing the empty pSE2, 7942AB-HAH6 and 7942B-HAH6 vectors were used. A total of 30 µg total protein of each fraction per lane was separated by SDS-PAGE and subjected to Western blotting. Proteins were detected with the antibody cocktail of the SbtA antibody and the anti-HA antibody. * = SbtA monomer; # = SbtB monomer; ## = possible dimer of SbtB. (TIF) [file pone.0115905.s002.tif]

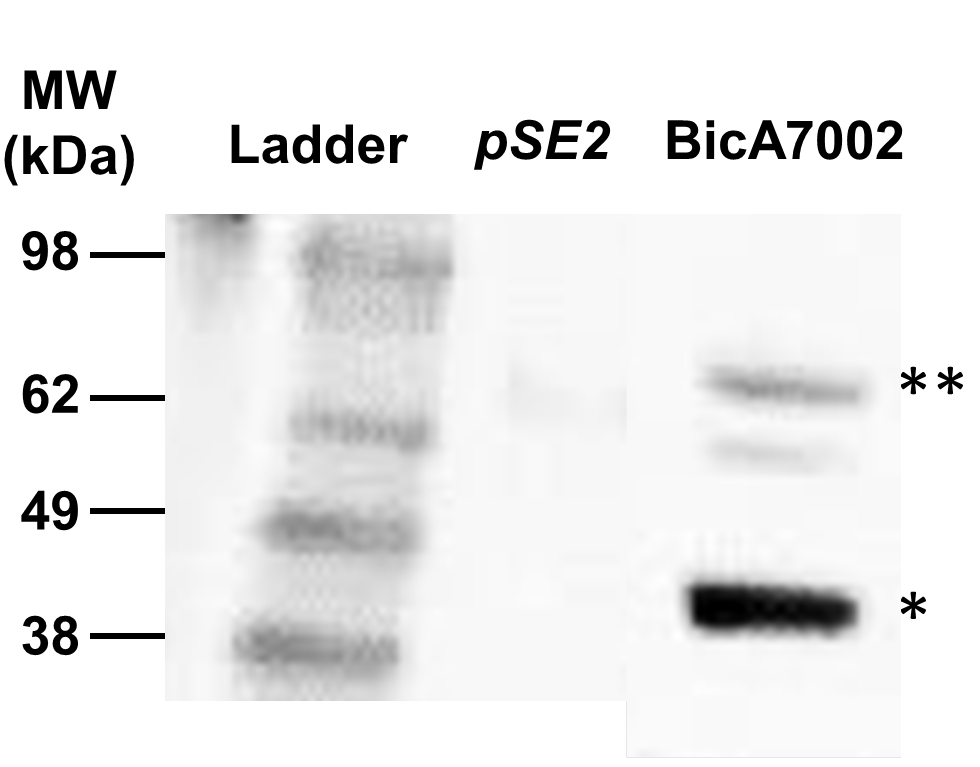

Supplement: S3 Fig — Detection of BicA7002 protein in the plasma membrane of E. coli by western blotting. Gene expression was induced for 2.5 h with 1 mM IPTG. The membrane-enriched protein fractions of E. coli containing the empty pSE2 and BicA7002 vectors were used. A total of 30 µg total protein of each fraction per lane was separated by SDS-PAGE and subjected to Western blotting. Proteins were detected with the antibody targeting the STAS domain of BicA. * = BicA monomer; ** = possible dimer of BicA. (TIF) [file pone.0115905.s003.tif]

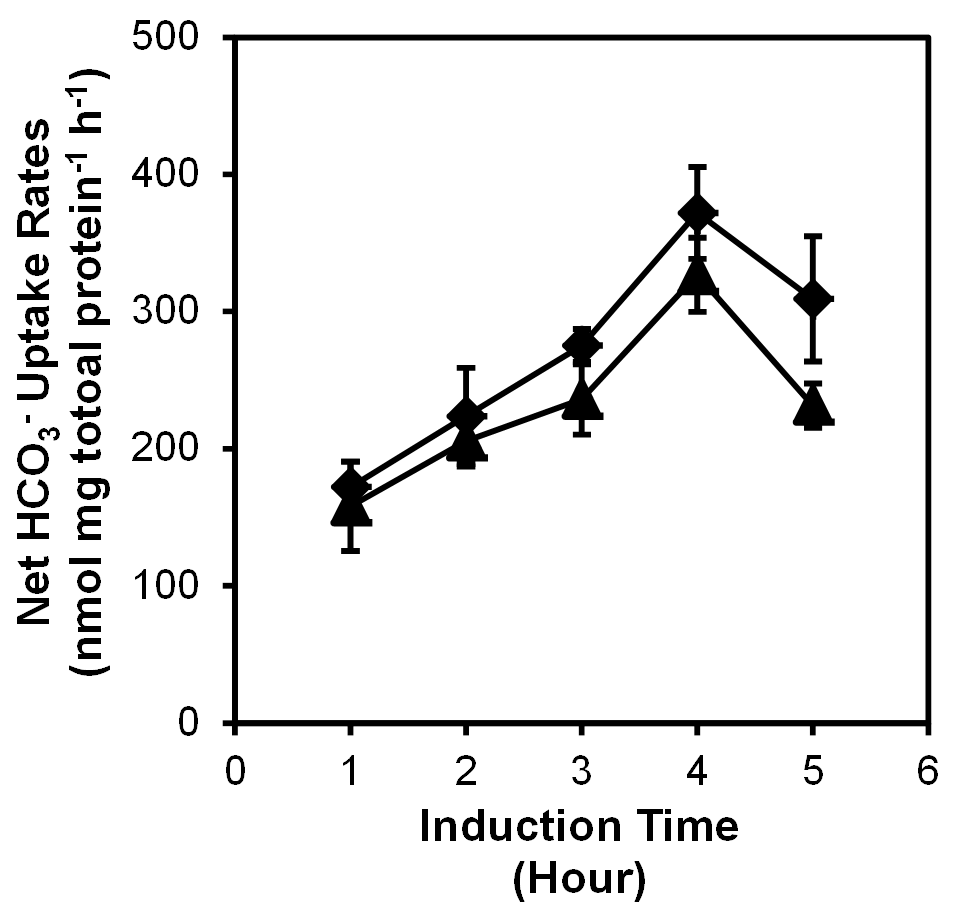

Supplement: S4 Fig — Optimisation of the induction time required for expression of SbtA7942 and SbtA7001. Cultures were prepared as described in Materials and Methods. Expression of SbtA7942 (diamond) and SbtA7001 (triangle) was induced by adding IPTG (1 mM) for up to 5 hours with samples taken every hour to determine uptake rates. Uptake experiments were performed in the presence of 50 mM NaCl and 50 µM H14CO3 -. Net uptake was calculated by subtracting data of pSE2empty vector (25∼30 nmol mg total protein−1 hour−1) from raw data for each transporter. Values in the figure are means ± SD (n = 6). (TIF) [file pone.0115905.s004.tif]

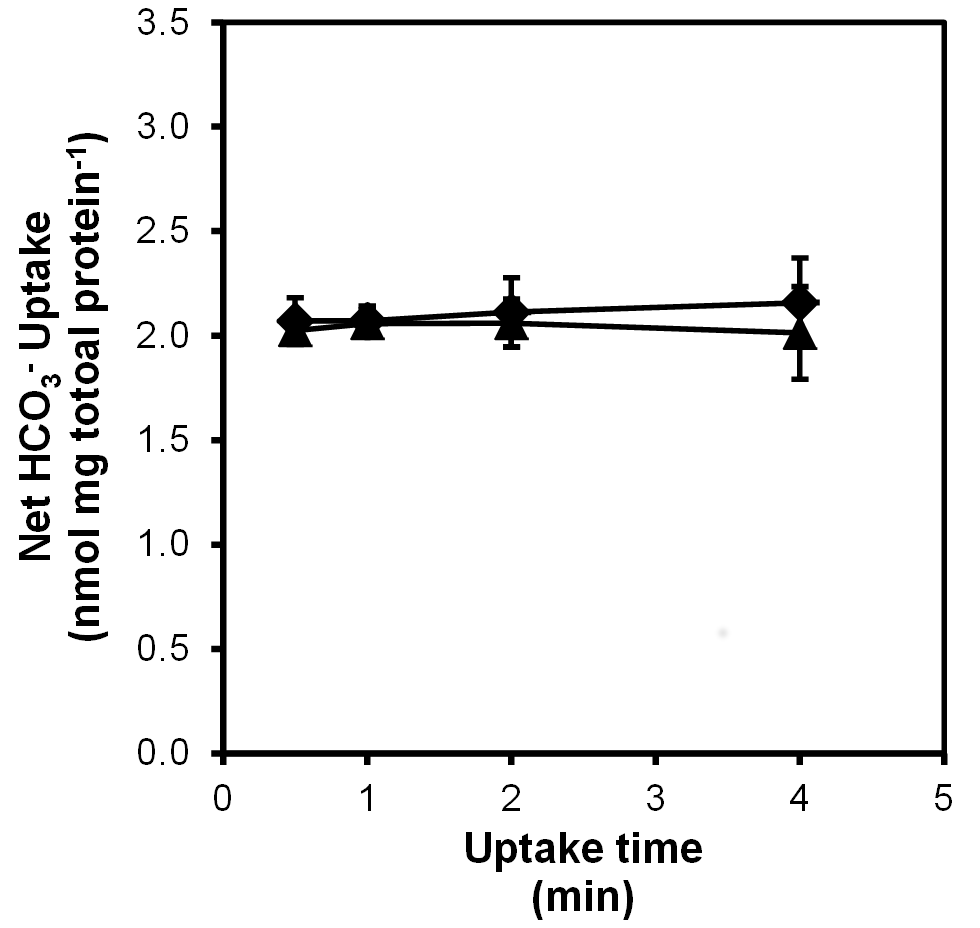

Supplement: S5 Fig — Uptake time course for SbtA7942 and SbtA7001. Cultures were prepared as described in Materials and Methods. Uptake experiments were done in the presence of 50 mM NaCl and 50 µM H14CO3 -. Cells were incubated with H14CO3 - for 0.5, 1, 2 and 4 mins. Net uptake was calculated by subtracting data of pSE2empty control (0.38∼0.55 nmol mg total protein−1) from raw data of SbtA7942 (diamond) and SbtA7001 (triangle). Values in the figure are means ± SD (n = 6). (TIF) [file pone.0115905.s005.tif]

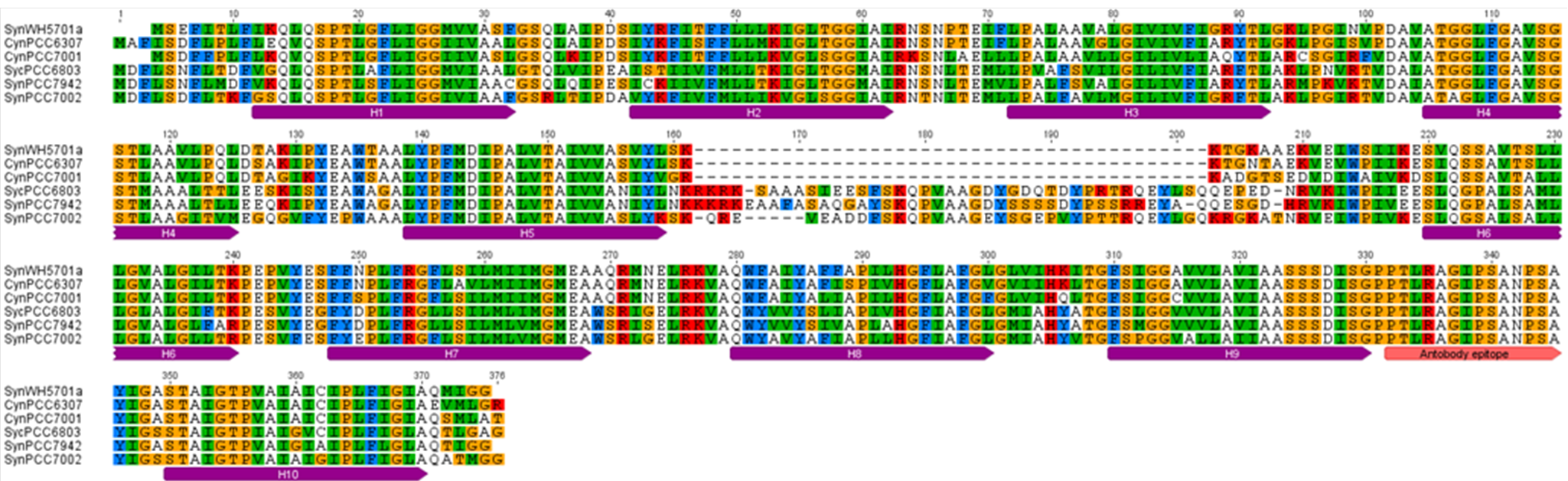

Supplement: S6 Fig — An alignment of the six SbtA forms used in the present study. The clones from β-cyanobacteria were Synechococcus elongatus sp. PCC7942 (SynPCC7942; freshwater), Synechococcus elongatus sp. PCC7002 (SynPCC7002; coastal/estuarine) and Synechocystis sp. PCC6803 (SycPCC6803; freshwater). The clones from α-cyanobacterial transitions strains were from Cyanobium spp. PCC6307 (CynPCC6307) and PCC7001 (CynPCC7001) and from Synechococcus WH5701 (SynWH5701). The positions of the membrane helices previously determined for Synechocystis PCC6803 SbtA are shown in purple. The conserved epitope region used for raising an antibody is shown in red. Residues are shaded according the functional categories: hydrophobic (green), positively charged (red), polar (orange) and aromatic (blue). (TIF) [file pone.0115905.s006.tif]
